# Screening for Dementia Caused by Modifiable Lifestyle Choices Using Hybrid PET/MRI

**DOI:** 10.3233/ADR-180098

**Published:** 2019-02-04

**Authors:** Frank S. Prato, William F. Pavlosky, Steven C. Foster, Jonathan D. Thiessen, Roderic P. Beaujot

**Affiliations:** a Department of Medical Biophysics, Western University, London, ON, Canada; b Lawson Health Research Institute, London, ON, Canada; c Department of Medical Imaging, Western University, London, ON, Canada; d Department of Sociology, Western University, London, ON, Canada

**Keywords:** Brain PET, dementia demographics, dementia screening, hybrid PET/MRI, lifestyle risks, magnetic resonance imaging, MRI-compatible PET, positron emission tomography

## Abstract

Significant advances in positron emission tomography (PET) and magnetic resonance imaging (MRI) brain imaging in the early detection of dementia indicate that hybrid PET/MRI would be an effective tool to screen for dementia in the population living with lifestyle risk factors. Here we investigate the associated costs and benefits along with the needed imaging infrastructure. A demographic analysis determined the prevalence of dementia and its incidence. The expected value of the screening program was calculated assuming a sensitivity and specificity of 0.9, a prevalence of 0.1, a QALY factor of 0.348, a willingness to pay $114,000 CAD and the cost per PET/MRI scan of $2,000 CAD. It was assumed that each head PET/MRI could screen 3,000 individuals per year. The prevalence of dementia is increasing by almost two-fold every 20 years due to the increased population at ages where dementia is more prevalent. It has been shown that a five-year delay in the incidence of dementia would decrease the prevalence by some 45%. In Canada, a five-year delay corresponds to a health care savings of $27,000 CAD per subject per year. The expected value for screening was estimated at $23,745 CAD. The number of subjects to be screened per year in Canada, USA, and China between 60 and 79 was 11,405,000. The corresponding number of head-only hybrid PET/MRI systems needed is 3,800. A brain PET/MRI screening program is financially justifiable with respect to health care costs and justifies the continuing development of MRI compatible brain PET technology.

## INTRODUCTION

An in-depth analysis of the demographics of dementia and a review of hybrid positron emission tomography (PET)/magnetic resonance imaging (MRI) predicts a future market that would justify the commercial development of a high-resolution brain PET system that could be inserted into a whole-body MRI to enable screening for dementia caused by modifiable lifestyle choices.

### Technology development and first commercial systems

Hybrid PET/MRI visionaries and pioneers first needed to develop technical solutions to allow PET systems to function in an MRI environment. The key development was the replacement of photo-multiplier tubes with avalanche photo-diodes or silicon photo-multiplier tubes [1]. As well there was a need to miniaturize components, so the entire PET detection system could be incorporated into a 5 cm thick sleeve inserted into a 70 cm diameter bore MRI [2]. Prior to the first wave of commercialization, which was undertaken by Siemens, Siemens developed an MR compatible head PET insert using avalanche photo-diodes [3]. However, only a few were manufactured and then commercially abandoned in favor of a whole-body system with PET integrated into a 3T MRI system which was marketed in 2011 [2]. Subsequently, in 2016, GE entered the market with a competitive whole-body PET 3T MRI system which used silicon photo-multiplier tubes, providing coincidence timing resolution that allowed time-of-flight PET [4]. Clearly the market for clinical PET was, at the time, driven almost exclusively by body applications for oncology. Brain PET insert technology would have to wait for market demand. By 2018 there were approximately 150 whole-body hybrid PET/MRI systems worldwide.

These first systems by Siemens and GE have undergone additional refinements to a large extent carried out by the early adopters. For MRI the attenuation of the PET annihilation radiation by the radiofrequency (RF) coils positioned between the subject and the PET detectors had to be reduced with minimal loss of RF signal. PET-compatible RF coils are continuing to be optimized [5]. More challenging has been the development of MRI-derived PET attenuation correction with the goal to achieve equivalent or better attenuation maps as are currently achieved by computerized tomography (CT) in PET/CT platforms [6, 7]. This needed development has resulted in delays, which persist today, in the incorporation of PET/MRI technology in multi-center clinical trials (e.g., Alzheimer’s Disease Neuroimaging Initiative (ADNI) trials) as consistency with PET/CT databases could not be assured. However, by 2018 MRI-derived PET attenuation maps have become competitive with CT-derived maps with the potential of exceeding CT-derived maps in the future [7]. However, there remains a further need of uniformity of approach through commercial standardization and ease of use [8, 9].

### Next generation of commercial PET/MRI systems

Given the slower than anticipated sales of hybrid PET/MRI systems, next generation commercial hybrid platforms may see a cycle time that exceeds 10 years. This has resulted in the 3T MRI portion of the hybrid platforms falling behind when compared with currently available MR systems (e.g., RF channel number, gradient performance). Similarly, the PET system performance is starting to fall behind the PET performance realized in state-of-the-art PET/CT systems [4, 10, 11]. This may result in a further decline in the hybrid whole-body PET/MRI market as the primary focus of clinical PET is in oncology and the preference to use hybrid PET/MRI over PET/CT in a number of oncology applications (e.g., prostate, breast, head and neck, and colorectal [9, 12, 13]) will be reduced if the PET of PET/CT is superior to the PET in PET/MRI with respect to spatial resolution and sensitivity [4, 10, 11, 14]. However, an MRI-compatible brain PET insert would be an effective tool in allowing the latest MRI technology to be used and defining a preferred hybrid modality use over PET/CT since brain clinical applications (such as epilepsy [15, 16]) and clinical trials (such as ADNI [17]) have demonstrated the importance of registering 3T MRI brain images to brain PET due to the improved soft tissue contrast of brain MRI over brain CT. Further the smaller diameter of a head PET system compared to a whole-body PET system has the potential to increase voxel resolution approximately 10 times, improve sensitivity approximately a factor of two, improve partial volume correction and improve motion correction [18]. As well PET/CT followed by 3T MRI requires two patient visits while only one is needed when hybrid PET/MRI is used. This clearly reduces cost, lowers the overall radiation dose to the patient as the CT procedure is avoided and improves subject compliance in clinical trials.

### Justification of an MRI-compatible brain PET

The justification to develop a MRI-compatible brain PET that could be delivered in late 2020 is driven by: 1) a shift in population demographics to a larger older fraction, 2) an increased lifespan accompanied by a significant increase in the fraction of the population suffering from neurodegeneration disease, 3) although the inevitable discovery of therapies that will slow or cure one or more of these diseases is still estimated to be 5–10 years away, it is known that approximately 50% of dementia can be prevented or delayed by modifying lifestyle [19–24], and 4) the continuous development of PET ligands that can detect biomarkers of neurodegeneration disease [25–27] (Pubmed “pet AND first in human studies AND brain” shows 142 publications from 2014 – 2017).

### Outline of our argument

Commercialization of MRI-compatible brain PET will depend on projected market demand (i.e., number of images per year) and a positive cost benefits analysis. We will: 1) estimate demand based on a demographic analysis of the prevalence and incidence of dementia (other applications including epilepsy, mental illness, stroke, traumatic brain injury, and brain research would further increase demand), 2) make a case that optimizing the PET resolution and sensitivity are important for head imaging, and 3) argue that it is unlikely that disruptive non-imaging technology would eliminate the need for a brain PET imaging as a screening tool.

## DEMOGRAPHICS OF DEMENTIA

### Population aging and rapid growth of the elderly population

If the 20th century was especially known for population growth, bringing the world population from 1.6 to 6.1 billion (a 3.8-fold increase), the 21st century will be one of marked population aging and high growth of the older population. While aging was already occurring in the 20th century, the dynamics of aging are now much different, bringing a marked increase in the size of the older population. The first phase of aging can be called “aging at the bottom” because it is due to a decline in fertility, and thus in the relative smaller size of the population at the bottom of the age pyramid [28, 29]. The aging that we are now seeing is an “aging at the top” with 1) a movement of the larger pre-fertility-decline cohorts into older ages, and 2) increasing life spans to the benefit of older segments of the population. In population projections that were made in the 1970s, it was often assumed that improvements in life expectancy would reach a plateau, as there would eventually be little room for improvements in mortality rates in infants, children and younger adults. However, this assumption has proven false as the elderly population is undergoing marked decline in age-specific mortality.

The medium projections of the United Nations (2017) are expecting a 32.4% increase in the world population, from 7.4 billion in 2015 to 9.8 billion in 2050. However, during this period, the population aged 65+ is expected to increase 2.5-fold, from 0.6 billion in 2015 to 1.5 billion in 2050. Over the period 2010 to 2030, the world population is expected to increase by 22.9% but the population aged 60 and over will increase by 82.8%, and the population aged 80 and over by 89.4% (Table 1). Over this 20-year period, the population aged 80+ in China will increase from 18.8 million to 40.8 million (2.2-fold) and in Canada from 1.3 million to 2.6 million (1.9-fold). China is undergoing a particularly rapid increase in the population aged 80+, from 22 million in 2015 to 121 million in 2050, or a 5.4-fold increase [30].

**Table 1:** Population (thousands) for world regions and specific countries, Total, Aged 60+, Aged 80+, 2010 and 2030

Area/country	Total			Aged 60 +			Aged 80 +		
	2010	2030	2030/2010	2010	2030	2030/2010	2010	2030	2030/2010
World	6,958,169	8,551,199	1.229	769,413	1,406,105	1.828	106,575	201,868	1.894
High-income countries	1,148,592	1,249,896	1.088	241,336	359,030	1.488	47,618	83,047	1.744
Other	5,809,577	7,301,302	1.257	528,077	1,047,075	1.983	58,958	118,821	2.015
China	1,359,755	1,441,182	1.060	171,120	361,620	2.113	18,777	40,843	2.175
Canada	34,169	40,618	1.189	6,819	11,849	1.738	1,345	2,606	1.937
USA	308,641	354,712	1.149	56,707	91,720	1.617	11,170	19,274	1.726

Notes: High Income: Countries classified by the World Bank as having 2016 per capita GNI of $12,336 or more. Other: World minus High Income. Source: United Nations, Department of Economic and Social Affairs, Population Division (2017). World Population Prospects: The 2017 Revision, custom data acquired via website.

### Other demands for brain PET imaging

Although here we will focus on a rationalization of brain PET in dementia, it is important to point out that there is growing need for brain PET imaging for several other conditions including: 1) mental illness (one in five North Americans experiencing a mental illness in their lifetime [31]), 2) traumatic brain injury (estimated at 69 million per year worldwide [32]) and 3) mild traumatic brain injury estimated at 42 million per year worldwide [33, 34]).

Further, proven clinical applications of hybrid brain PET/MRI as in epilepsy would clearly benefit from increased spatial resolution [35].

### Rapid increase in persons living with dementia

The projections of persons living with dementia are based on population projections and the rates of dementia at various ages. Alzheimer’s Disease International (ADI) [36] has become a reliable source for these projections for regions of the world. Following criteria currently used in clinical practice and epidemiological studies, ADI defines dementia according to DSM-IV or ICD-10 criteria, or similar pre-existing clinical criteria. The ADI projections have been updated in the *World Alzheimer Report 2015: The Global Impact of Dementia*—*An analysis of prevalence, incidence, cost and trends*. To establish the rates, Martin Prince and his colleagues [37] first undertake a meta-analysis of various studies that seek to establish age-specific rates (WAR 2015:10–28 [37]). In particular, the meta-analysis excludes studies where the sample had been drawn from specialized care institutions or where the sample is not representative. That is, the ADI estimates rely on studies conducted in the population, whether or not they are accessing services. The rates of dementia prevalence are based on 273 population-based studies across the regions of the world. Once the rates are established, they are applied to the population projections of the United Nations. Table 2 provides the results showing the persons living with dementia across the world, and in the specific countries of China, Canada, and the United States (US) for 2010 to 2050. At the world level, these estimates show 40.1 million people with dementia in 2010, rising to 74.7 million in 2030, and to 131.5 million in 2050. This represents close to a doubling of persons with dementia every 20 years.

**Table 2:** Prevalence of dementia (thousands) for world regions and specific countries, 2010–2050

Area/country	2010	2015	2020	2025	2030	2035	2040	2045	2050
World	40,121	46,780	54,266	63,454	74,689	87,880	102,151	116,778	131,454
High-Income countries	17,028	19,502	21,965	24,733	27,951	31,716	35,706	39,143	42,177
Other	23,093	27,278	32,301	38,721	46,738	56,164	66,445	77,636	89,278
China	8,146	9,518	11,118	13,322	16,184	19,358	22,290	25,132	27,856
Canada	483	556	639	748	886	1,056	1,221	1,349	1,452
USA	3,760	4,227	4,778	5,468	6,397	7,513	8,661	9,597	10,285

Notes: High Income: Countries classified by the World Bank as having 2015 per capita GNI of $12,476 or more. Venezuela and Equatorial Guinea are classified as High Income in 2015 but as Middle income in 2016 (Table 1). Other: Sum of Low Income and Middle Income countries. Source: Alzheimer’s Disease International, 2015. World Alzheimer Report 2015, p. 8, plus special tabulations obtained from, Maëlenn Guerchet of the Center for Global Mental Health at King’s College London on 23 April 2018 and 17 May 2018.

For the period 2015–2030, the total persons living with dementia would increase by 60% for the world as a whole, by 70% for China, 59% for Canada, and 51% for the US.

The World Alzheimer Report 2016 includes a valuable Chapter 6 entitled Dementia care in Canada, China, Indonesia, Mexico, South Africa, South Korea, and Switzerland [30]. For Canada, the projections are based on the prevalence rates of the Canadian Study of Health and Aging (1994). A review posted on the web site of the Public Health Agency of Canada confirms that this 1994 Canadian Study of Health and Aging provides the best and most reliable population data source for present and future estimates of dementia [38]. Based on these prevalence rates, the number of people with dementia in Canada is expected to increase from just over 556,000 in 2015 to 886,000 in 2030 (see Table 2). This chapter also observes that, in 2015, 65% of the 556,000 cases were women and nearly half were aged 85 or more. For China, this Chapter 6 quotes the figure of 9.5 million people with dementia in 2015, comprising 20% of the total number of people in the world with dementia, rising to over 16 million by 2030 [30].

ADI also projects the incidence or new cases of dementia for various world regions. These projections are based on a meta-analysis of 46 studies permitting separate estimates of incidence rates for the world and six regions [37]. For all studies combined, the incidence of dementia doubles with every 6.3-year increase in age, from 3.9 per 1000 person years at age 60–64 to 104.8 at age 90+. In estimating the annual incident cases, ADI first estimates the numbers at risk (total population in each age group minus numbers with prevalent dementia) and multiples this by the appropriate incidence rate. In 2015, the global incidence is estimated at 9.9 million, which is slightly more than one-fifth of the global prevalence [37].

In Table 3, we have used the ADI estimation procedure to calculate the incidence for China, Canada, and the US in 2010, 2020, and 2030. The results are shown separately for ages 60–79 and for ages 60 and over. Over the period 2010–2030, the annual incidence for the 60+ is found to increase by a factor of 2.1 for China, 1.9 for Canada, and 1.7 for the US with very similar increases for the 60–79 cohort. In 2020, the incidence in the 60+ cohort is estimated at 2,365,000 for China, 131,000 for Canada and 1,045,000 for the US. These represent for the 60+ 21.3%, 20.5% and 21.9% respectively of the prevalence levels of these countries in 2020.

**Table 3:** Incidence of dementia (thousands) for world regions and specific countries, 2010–2030

Area/country	Aged 60–79			Aged 60+		
	2010	2020	2030	2010	2020	2030
World^*^	5,470	7,247	10,083	10,124	13,927	19,195
High-Income countries	1,622	2,014	2,440	4,046	5,302	6,813
Other	4,010	5,523	7,981	5,726	8,082	11,523
China^**^	1,195	1,709	2,542	1,636	2,365	3,512
Canada^**^	43	62	81	95	131	183
United States of America^**^	363	510	645	813	1,045	1,412

Note: ^*^The incidence of dementia was obtained by multiplying rates at age groups 60–64 to 90 + by the corresponding populations. ^**^The incidence of dementia was obtained by first obtaining the population at risk (total population minus persons who already have dementia) then multiplying the incidence rates at age groups 60–64 to 90 + by the corresponding populations at risk. Source: See Table 1 for source of population data. Prevalence and Incidence rates were taken from Alzheimer’s Disease International, 2015. World Alzheimer Report 2015, p. 20 and p. 33. The rates for East Asia were used for China and those for North America were used for USA. For Canada, the prevalence rates were taken from a personal communication with Maëlenn Guerchet of King’s College London dated 1 June 2018, and the incidence rates for North America were used.

## A MICRO-SIMULATION FOR CANADA: PREVALENCE, INCIDENCE, COSTS, AND IMPACT OF DELAYING ONSET FOR FIVE YEARS

Most projections of the prevalence of dementia, including those of ADI [36], are obtained by macro-simulation, where population projections by age are multiplied by age-specific prevalence of the disease. An important study at Statistics Canada has used, instead, a population-based longitudinal micro-simulation approach [39, 40]. This starts with the census population, including each person’s characteristics, and projects each person forward year-by-year as they are exposed to various risks, until their departure from the population (death or emigration). This methodology allows for differential risks, to give birth, migrate, or die, based on the individual’s characteristics. In projecting dementia, the model begins with individuals with dementia and without dementia. As they are moved forward year-by-year, persons with dementia are exposed to dementia-specific mortality risks. For persons without the disease, the incident cases of dementia are projected based on risk-factors.

Manuel et al. [39] define dementia as cases identified through physician-coded diagnosis in health administrative data. This is a narrower definition than that used by ADI where population estimates include cases that have not been diagnosed by physicians providing care. The ADI estimates, based on the 1994 Canadian Study of Health and Aging, show 483,000 persons with dementia in 2010, while the diagnosed cases in 2011 add up to 340,000 [39], suggesting 30% of persons with dementia in Canada are under diagnosed [39].

The projections show a doubling in the prevalence of dementia, from 340,000 people in 2011 to 674,000 in 2031 [39]. Part of the increased prevalence is due to the projected reductions in all-cause mortality and dementia mortality over time. The authors cite Barnes and Yaffe [24] who estimate that 50% of Alzheimer’s disease is attributable to diabetes, hypertension, obesity, depression, physical activity, smoking, and low education. While the model projects all of these risk factors except depression, the authors decided that the predictive nature of these risk factors on dementia incidence remains poorly understood. Thus, they only used age and sex as risk factors. These incidence rates start at ages 40–44, where the rate is 0.110 per 1000 women and 0.226 per 1000 men. These rates rise to 5.547 and 5.921 per 1000 women and men, respectively, at ages 70–74. At ages over 75, the incidence rates are higher for women than men peaking at 48.040 and 43.578 per 1000, respectively, at age group 95+ [39].

Besides projecting the prevalence of dementia, Manuel et al. [39] undertake a sensitivity analysis where they model a five-year delay in the age-specific incidence of dementia. In the baseline scenario, where the incidence rates are held constant, the number of people with dementia increases by a factor of 1.98 between 2011 and 2031 (from 340,000 to 674,000 cases). In the scenario with a five-year delay, the increase is by a factor of 1.10 or 10% (from 340,000 people in 2011 to 374,000 in 2031). The number of people receiving care would increase by a factor of 1.11 (from 261,000 in 2011 to 291,000 in 2031) rather than the factor of 2.00 of the baseline scenario (from 261,000 in 2011 to 522,000 in 2031). This corresponds to a decrease of prevalence of 45%. It is of interest to note that Brookmeyer et al. [41] predict that a 5-year delay would reduce prevalence in the US by half while the analysis of Jorm et al. [42] for Australia suggest a reduction by 44%. A five-year delay in the age-specific incidence of dementia would reduce the total cost to the Canadian health care sector by $8 billion: the total costs in 2011 are estimated at $9.2 billion, while in 2031 these total costs are estimated to increase to $18.2 billion under the baseline scenario and $10.25 billion under the scenario of a five-year delay in the incidence of dementia [39].

## ANALYSIS OF IMPACT OF A FIVE-YEAR DELAY IN DEMENTIA ONSET

Manuel et al. [39] estimates the direct savings at $8 billion per year for a five-year delay in onset. This corresponds to a savings of approximately $27,000 per year per person and over the five years $135,000 per person. In simple terms, if the direct cost of doing a hybrid brain PET/MRI was $2,000/study and one had to screen approximately 10 patients with higher risk to develop dementia due to lifestyle in order to detect one that did not, at the time, have any evidence of cognitive impairment then direct costs of imaging would be $20,000, resulting in a net savings of $115,000 per person if for all detected patients, lifestyle changes and treatment resulted in a 5-year delay in onset. Of course, both the true positives and false positives would be identified and reduce savings accordingly. Note that the current Anti-Amyloid Treatment in Asymptomatic Alzheimer’s study (“A4 study” sponsored by NIH and Eli Lilly and Company) supports this one in ten screening factor for a study prevalence of 0.1 (*p* = 0.1). It may be reasonable to argue for a delay of onset by five years just based on analysis of risk factors. Barnes et al. [24] estimates seven risk factors contribute to half of Alzheimer’s disease cases globally. Such risk factors can help identify those to be screened and since these risk factors, including physical inactivity, smoking and obesity, can be modified, early evidence of future dementia prior to cognitive decline could have an impact on patient motivation [43] that would delay onset [44, 45]. Note that the prevalence in the population to be screened could be further increased by taking other risk factors into account such as genetic predisposition. For example the presence of APOE3/E4 allele combination is believed to account for 70% of the risk of Alzheimer’s disease [46], but effective therapies for these other risk factors that would delay onset still need to be developed [47, 48]. However, evidence is starting to accumulate suggesting that physical activity [20] or general lifestyle changes [49] can benefit older at risk individuals even in the presence of APOE4. Note that a health intervention program for risk reduction in dementia has been predicted to be cost effective in the Swedish/Finnish setting [44].

We next make an estimate of the utility of a PET/MRI brain scan by making a number of assumptions: 1) prevalence of the population to be screened of *p* = 0.1 (which is consistent with the experience of screening for amyloid deposition with PET in the A4 study; [50]), 2) test sensitivity (Se) is 0.9, test specificity (Sp) is 0.9, 3) the direct cost of the test is $2,000, and 4) the delay in onset is 5 years. The value of each year of delay in onset will be estimated using the quality-adjusted life years (QALY) formulation. In a Swedish/Finnish population Zhang et al. [44] estimated the difference in QALY between non-demented and demented to be 0.348 of the Willingness To Pay which in 2011 was estimated to be $80,000 USD. If we assume inflation at 2% per year and Canadian exchange of $1.30 per USD, then 0.348 QALY is equal to $80,000×1.10 (inflation)×1.3×0.348 or $39,811 CAD.

The cost of therapy can vary from approximately $1,000 CAD for a multi domain health promotion five-year program [24, 44] up to approximately $400 CAD/month (current cost of brand name drugs for dementia therapy are all under $400/month) hence we have used $4,800/y or $24,000 total for the five years as a reasonable upper limit estimate.

Given these assumptions and using values of the utility matrixes (U) reported in (Table 4), the expected value (E) of a PET/MRI brain imaging test for dementia can be calculated as [51]:

$$\begin{array}{ccc} \;\;\;\;\;\;\;\;\;\;\;\;\;\;\;\;E\; & & =\;U\left(\mathrm{TP}\right)\;\times \;\mathrm{TPF}\;\times \;p\;+\;U\;\left(\mathrm{FP}\right)\;\times \;\mathrm{FPF}\;\times \;\left(1-p\right)\;+\;U\left(\mathrm{TN}\right)\;\times \;\mathrm{TNF}\;\times \;\left(1\;-\;p\right)\;+\;U\left(\mathrm{FN}\right)\;\times \;\mathrm{FNF}\;\times \;p\\ & & =\;308,056\;\times \;0.9\;\times \;0.1\;-\;\$26,000\;\times \;0.1\;\times \;0.9\;-\;\$2,000\;\times \;0.9\;\times \;0.9\;-\;\$2,000\;\times \;0.1\;\times \;0.1\\ & & =\;\$23,745 \end{array}$$

**Table 4:** Estimate of utility matrix for TP, FP, TN and FN

U (TP)	= 135,000 (savings of 27,000 per year^1^ per subject for 5 years)
	+ 199,056 (QALY^2^ set at 39,811 and summed over 5 years)
	–24,000 (cost of therapy^3^ total over 5 years)
	–2,000 (cost of PET). = 308,056
U (FP)	=–$24,000 (cost of therapy)
	–2,000 = –26,000
U (TN)	=–2,000 (cost of PET)
U (FN)	=–2,000 (cost of PET)

^1^The $27,000 per year savings were estimated from Manuel et al. [39] where a savings of 8 billion was estimated if incidence was reduced by 45% by delaying onset for 5 years. ^2^QALY difference between non-demented and demented set at 0.348 which was valued at $39,811 CAD (Zhang et al. [44]). ^3^The cost of therapy can vary from approximately $1,000 CAD for a multi domain health promotion five year program [24, 44] up to approximately $400/month (current cost of brand name drugs for dementia therapy are all under $400/month) hence we have used $4,800/y or $24,000 total for the five years as a reasonable upper limit estimate.

Where TPF represents True Positive Fraction, FPF represents False Positive Fraction, TNF represents True Negative Fraction and FNF represents the False Negative Fraction. These are related to the Se and Sp as: TPF = Se, FPF = (1–Sp), TNF = Sp and FNF = (1–Se).

We have used an estimate of 0.9 for both the Se and Sp. In general, diagnostic imaging tests rarely exceed these values. However, given the Se and Sp of the first generation of brain PET probes used in multi-center trials, such as ^18^F-labeled amyloid-β, this estimate for next generation PET probes seems reasonable. For example, Sabri et al. [52] give values for Florbetapir of 98% for Se and 89% for Sp and Clark et al. [53] give values of 92% (Se) and 100% (Sp) when compared to autopsy material. In a recent meta-analysis, amyloid PET yielded a pooled Se of 90% and Sp of 85% for distinguishing patients with Alzheimer’s disease from healthy controls [54]. In a different meta-analysis, it was 83% (Se) and 89% (Sp) for MRI [55]. It is reasonable to predict a PET/MRI test combining information from both modalities and using a next-generation brain PET probe which could equal or exceed our estimated Se and Sp [56]. There is less data on detection years before evidence of clinical symptoms; however, studies that have been done on populations with familial dementia suggest detection of amyloid-β at least 10 years prior to cognitive decline with Se and Sp in the range of 0.9 [57]. Note that these values of Se and Sp relate to PET or MRI alone and the use of combined PET/MRI would further improve the Se and Sp values [58–60] particularly for the early detection of vascular dementia and Alzheimer’s disease [61, 62].

The calculation of the expected value could be broken down and values of Se, Sp, and p substituted for atrophy by MRI, vascular deregulation by PET alone (e.g., ^15^O2-water) or MRI alone (e.g., arterial spin labeling or ASL), glucose-PET alone, amyloid-PET alone, or tau-PET alone, but the current state of knowledge regarding progression for the onset of late onset dementia remains controversial. For example, the progression model suggested by Jack et al. [58] predicting changes in brain amyloid and tau protein accumulation prior to symptoms has been seriously challenged by the work of Iturria-Medina [62] who provide evidence that the first change seen is to vascular dysregulation as measured by ASL-MRI and that symptoms occur at approximately the same time as there are changes to these biomarkers. Nevertheless discoveries continue as to how PET and MRI can be combined to track early signs of Alzheimer’s disease [63] and how deep learning as applied to glucose-PET can be used to make the correct diagnosis 75.8 months earlier than is possible using conventional approaches to diagnosis [64]. Instead, we have assumed that the most expensive imaging technology (hybrid PET/MRI) will need to be used and combining that cost with best estimates from the literature (such as QALY) and relatively high estimates of cost for other aspects such as therapy we have shown a net positive utility. As dementia screening through imaging is cost effective continuing research into imaging biomarker discovery is justifiable.

We have estimated the cost per PET/MRI imaging session to be $2,000 CAD, significantly lower than the current cost of amyloid PET scans, which are estimated to be as high as 5,000 CAD in the US [65]. However, most of the cost of the PET probe is fixed (approximately $3,000 CAD) and in a screening scenario would be reduced by dividing the cost of the PET probe production run by the number of patients screened, per production run and hence resulting in an estimate of $500 CAD per imaging session, if 10 patients could be imaged from one production run. The cost of the hybrid PET/MRI exam, including operating costs and depreciation would be reduced if a dedicated brain PET insert was developed and sold at a price of $1.3 million CAD (rather than $6 million CAD for a whole-body hybrid system) and be inserted into a 3T MRI system valued at $3 million CAD). If we depreciated straight line over 10 years, assume a service cost of 10%, operating costs of $100 CAD per examination, physician interpretation cost of $250 CAD per examination and 3,000 studies per scanner per year, we see that a cost of $2,000 CAD per PET/MRI imaging procedure is in fact a conservative estimate ($500 +  ($4,300,000×0.1)/3,000 +  ($4,300,000×0.1)/3,000 + $100 + $250 = $1,136 CAD). The additional $864 CAD allows for a buffer of unspecified costs difficult to quantify at this time such as development of PET/MRI integration software and the extent of the needed nuclear medicine infrastructure to be placed in an MRI facility. Note that $864 CAD per procedure if attributed to equipment and laboratory infrastructure depreciated straight line over 10 years corresponds to approximately $26 million CAD, i.e., $864×3,000 examinations per year× 10 years.

Weimer and Sager [66] have argued that early diagnosis and treatment of dementia is not only fiscally attractive from both state and federal perspectives but also socially desirable in terms of increasing economic efficiency: “failure to fund effective caregiver interventions may be fiscally unsound”.

## ESTIMATE OF NUMBER OF IMAGING PROCEDURES PER YEAR

The analysis in the section immediately above suggests cost effectiveness under the given assumptions for an individual subject. What is now needed is justification for the development and manufacture of an MRI-compatible brain PET that will be needed to reduce screening costs and dramatically improve voxel resolution. Market size is dependent on the number of subjects to be scanned per year. This can be estimated under the following assumptions:

1) We propose two analyses: one to screen the population between 60 and 79 and a second for all over 60, i.e., 60 + . Initiation of screening at age 60 will give time to modify lifestyle risks provided hybrid PET/MRI can identify, approximately 10 years in advance, those who will become cognitively impaired. This explicitly excludes early onset disease which tends not to be due to lifestyle risks [57].
2) We propose to only screen those that are not cognitively impaired since there is strong evidence that therapies are ineffective once there is even mild cognitive impairment [67].
3) We propose to only screen that fraction of the population that is at risk due to lifestyle risk factors which are estimated to cause 50% of the incidence of dementia [24, 44].
4) We estimate the number of patients to be screened i) to be equal to the risk factor for dementia between 60 and 79 and 60+, ii) to be divided by two to include only those subjects with modifiable lifestyle risk factors, and iii) to be multiplied by ten assuming a prevalence of those that will develop dementia in the tested population of 10% (as has been demonstrated in the A4 study).

With these assumptions we estimate, using the incidence values reported in (Table 3), the number to be screened in Canada, the US, and China in 2020 (see Table 5). How does this translate to the number of brain PET inserts that the market would support? It could be assumed that one brain PET unit has the capacity to do 4,000 procedures (over a 250-day work year this would be 16 procedures per day and at an estimated 1 hour per procedure would require at least two shipments of an ^18^F PET ligand). However, on average, the number will be somewhat lower given that some units will do less procedures as they will have limited PET probe delivery to cover extended hours and other units will service a catchment with less than the needed 4,000 procedures. (For example, in Canada if screening the 60–79-year cohort the catchment would be an estimated population of 440,000 if the population of the catchment had a demographic representative of the Canadian average.) In addition, for other units, the planned anticipated use to service a catchment will be much less with the associated MRI unit being used only for MRI when the brain PET is not in use. Hence, we have estimated the number of units assuming 3,000 procedures per year per unit. Table 5 indicates the number of units needed to screen patients once a year in Canada, the US, and China at between 3,801 (60–79) and 5,902 (60+). (Note that these three countries may only represent 50% of the world demand since, as shown in (Table 2), world prevalence exceeds by more than a factor of two the summed prevalence in China, Canada, and the US).

**Table 5:** Number of PET/MR Scanners Needed for Dementia Screening of 60–79 and 60 + starting in 2020

Country	Incidence/y^1^	Life Style Risk Factor^2^	Number to be screened^3^	Number of scanners needed^4^
Canada
60–79	62,000	31,000	310,000	103
60+	131,000	65,500	655,000	218
USA
60–79	510,000	255,000	2,550,000	850
60+	1,045,000	522,500	5,225,000	1,742
China
60–79	1,709,000	854,500	8,545,000	2,848
60+	2,365,000	1,182,500	11,825,000	3,942
Total Scanners for Canada, USA and China				3,801–5,902

^1^Incidence for Canada taken from Manuel et al. 2016 [39] multiplied by 1.3 to correct for cases not diagnosed in the medical setting. ^2^Assume 50% of dementia is caused by Life Style Risk Factors [24, 44]. ^3^Assume prevalence in the population to be screened is 10% [50]. ^4^Assume one scanner can do 3,000 procedures per year. ^5^USA incidence taken from the Canadian incidence [39] normalized to the expected difference in population in 2020 (334 versus 37.6 million) and corrected by relative prevalence. ^6^China incidence was taken from Canadian incidence normalized to the expected population differences in 2020 (1,403 versus 37.6 million) and corrected by the estimated relative prevalence in 2020 [39].

## EQUIPMENT SPECIFICATIONS

### Brain PET system sensitivity

Given the large numbers of patients that would need to be screened, it will be important to maximize sensitivity to reduce radiation dose. It is estimated that for a 35 cm bore diameter with an axial length of 25 cm, the sensitivity would be around two times greater than current whole-body PET/MRI systems. State-of-the-art PET systems have exceptional timing resolution (200 to 400 ps), allowing the system to place the location of each gamma ray more accurately based on its estimated time-of-flight (TOF) to each PET detector [4, 10, 11, 14]. This contributes to an effective gain in sensitivity dependent on both the timing resolution and object size, with a maximum TOF gain found in the center of the object. Sensitivity gain due to TOF information could be as much as 10× higher in the center of the brain using a TOF-capable, head-only PET/MRI with a 200 ps timing resolution (see Table 6) [11, 68]. Assuming the effective dose equivalent could be reduced by a factor of 7 from 7mSv to 1mSv [69], there would be a reduction in the number of induced cancers from 420 to 60 per million subjects screened assuming the linear no threshold model and a risk of 6×10^–2^ Sv^–1^ [70] which may overestimate the risk [71].

**Table 6:** Specifications for whole-body PET/MRI systems, head-only PET system, and theoretical head-only PET/MRI system

	Siemens Biograph mMR^1^	GE Signa PET/MR^2^	Siemens HRRT (PET Only)^3^	Head-Only PET/MRI^4^
Year	2011	2016	2002	2020
Bore/Axial FOV	60/25.6	60/25	35/25.2 cm	35/25 cm
Crystals (mm^3^)	4×4×20	4×5.3×25	2.1×2.1×15	2×2×20
Voxel Volume (mm^3^)	80	97	13	8
Timing Resolution (ps)	2930	390	Non-TOF	200
Sensitivity/TOF Gain	15 cps/kBq	21/45 cps/kBq	25 cps/kBq	30/125 cps/kBq

^1^Delso et al., 2011 [2]. ^2^Hsu et al., 2017 [4]. ^3^Wienhard et al., 2002 [89]. ^4^Estimated by extrapolation from existing state-of-the-art [11, 14] using medical physics principals presented in Moses 2011 [72] and Surti 2015 [68].

### Brain PET spatial resolution

Given theoretical limits in spatial resolution [72] and current high resolution whole-body PET currently being marketed in PET/CT systems, we estimate best resolution for a head unit with dimensions of 35 (bore) by 25 (axial) of approximately 2 mm FWHM (see Table 6). This estimate is supported by the specifications that have been attained by the latest generation of whole-body PET systems, i.e., a resolution of 3.23 mm FWHM (radially with 3.27 mm tangentially and 3.84 mm axially) [11, 14] for a ring diameter of 820 mm using 3.2 mm LSO crystals. So, using the formulation proposed by Moses [72] to scale down to a head-only system pixel volumes could be optimized at 8 mm^3^ as compared to a whole-body system with 36 mm^3^ (i.e., 3.23×3.27×3.38) or 450% better voxel tissue discrimination. Note that the simultaneous PET/MRI may allow partial volume correction of PET images using the higher resolution MRI data that, for 3T brain imaging, can easily achieve 1 mm^3^ voxel tissue resolution. It is unlikely that this significant partial volume correction can be achieved using the sequential technology of PET/CT or even sequential PET followed by MRI. This dramatic improvement in spatial resolution achieved with an optimized brain PET would allow smaller amounts of abnormal tissue to be detected, improving sensitivity and specificity of early disease detection. For example, there is growing evidence that amyloidosis often starts in the entorhinal cortex [73] with heterogeneous changes in volumes and amyloid-β/tau accumulation in the hippocampal subfields in preclinical Alzheimer’s disease [74–76]. This would also improve detection of non-diffuse disease as would be the case for the detection of small regions of epileptic focus in occult epilepsy [35] using ^18^FDG or disruptions of synaptic density in neurodegenerative diseases [27].

### Hybrid PET/MRI versus sequential PET/MRI versus “sequential” PET/CT

Hybrid PET/MRI that allows simultaneous acquisition over the brain would have a number of advantages over sequential approaches such as PET followed by MRI or PET/CT, since PET and CT are acquired sequentially as the patient is moved on the scanner bed between the PET and the CT acquisition. These advantages are:

1) A one “stop shop” replacing sequential PET/CT followed by MRI which reduces costs and would improve subject compliance.
2) The number and frequency of brain PET investigations would result in a significant dose from CT if the combination of PET/CT technology is used rather than PET/MRI. In an Australian study of 680,000 people exposed to one or more CT scans, overall cancer incidence due to the radiation exposure from CT was 24% greater in exposed than for an unexposed control group [77].
3) It has been shown in the ADNI that MRI adds considerably to the diagnosis as it is superior in brain soft tissue contrast compared to x-ray CT [17, 58–62].
4) MRI can detect presence of neuro-chemicals if their concentration is one millimolar or higher. Although PET is a billion times more sensitive it can only detect one ^18^F labeled ligand during a single examination. The use of MRI as a substitute for other PET ligands where neuro-chemical concentrations are high enough will allow a more thorough examination during a single patient imaging session. For example, functional MRI (fMRI) techniques such as arterial spin labeling could be used to measure brain blood flow as a surrogate for measuring glucose metabolism by PET allowing PET to be used to quantitate beta amyloid or tau burden [78].
5) Simultaneous PET and MRI of the brain adds to the value of the examination. This growing area will be very significant and will demonstrate that other approaches, such as sequential PET followed by MRI where the technology of PET is positioned just outside the entrance bore of the MRI, are not competitive [79]. Simultaneity allows a) the combination of fMRI and PET to determine the directionality of brain connections, which cannot be done without simultaneous acquisition [80], b) the potential to significantly lessen the invasiveness of many PET procedures by eliminating the use of arterial sampling [81, 82], and c) removal of motion artifact in the PET data [18].
6) Alternative imaging technologies: At this point in time, to the best of our knowledge, there are no alternative technologies to PET. MRI is, at times, proposed but it lacks sensitivity. High field MRI (e.g., 7 Tesla) provides submillimeter resolution but disease detection would depend on volumetric changes in the brain such as atrophy. There is mounting evidence that once disease specific atrophy is detected it is too late for the initiation of treatment for neurodegenerative conditions [22, 83, 84]. However, the use of 3T MRI to detect vascular dysregulation in a hybrid PET/MRI examination may be helpful in early detection [62] and further improve the Se and Sp of hybrid PET/MRI.
7) Alternate tests: Blood tests have dramatically improved in sensitivity although natural background of biomarkers currently limits the technology [85, 86]. Nevertheless, systemic detection seems to require imaging for localization. For example, a measurement of rising prostatic specific antigen (PSA is a blood test) has stimulated the discovery of PET probes for localization of disease, i.e., the development of PET ligands to the prostatic specific membrane antigen [12]. Hence, it is likely that blood tests will not be sufficiently specific for differentiation of brain diseases. Rather, when such blood tests are developed, they will further stimulate brain PET needs by identifying high risk individuals to be screened by brain PET, further improving efficacy of screening by increasing the incidence of the disease in the population to be screened [87, 88].

## IS THERE A BUSINESS CASE?

The current commercial competition for a future PET head insert is the currently available whole-body hybrid PET/MRI systems. Even if these systems continue to be equal to the combined cost of a 3T MRI and a PET/CT they are more efficient as they reduce patient visits from two to one. Also, once it is recognized that the issue of MRI-directed PET attenuation correction has been resolved [7–9], just avoiding the CT radiation dose will make PET/MRI the hybrid modality of choice for brain PET imaging. Currently the hybrid PET/MR market is growing slowly and the commercial leaders (Siemens and GE) may not be currently investing into the next generation of whole-body PET/MRI machines until the current market systems are ten or more years beyond their first introduction. Hence spatial resolution will remain for the foreseeable future at about 4.3 mm FWHM [4] for voxel sizes of 80 mm^3^ or 800% greater than a proposed head-only system (Table 6). Also, it may be that manufacturing of a head-only unit by any of the main manufacturers will not be initiated until total global sales of hybrid PET/MR systems reach between 75–100 per year. This leaves a window of about 5 years for the development of head PET inserts by academic centers and other small commercial companies.

In addition to the cost advantage that the combination of a stand-alone 3T MRI and a high-resolution MR-compatible PET insert would have over a whole-body PET/MRI, there are also other advantages of the head insert approach. Integrated hybrid PET/MRI systems result in a compromise to the MRI platform and this is related to the increased MRI bore size needed for whole-body hybrid PET/MRI (i.e., 70 cm versus 60 cm). A 70 cm diameter MRI bore will always have inferior gradient performance compared to a 60 cm diameter MRI bore. But there is one other major drawback to the integrated PET/MRI system. MRI technology continually evolves faster than PET technology and the MRI part of the hybrid PET/MR systems have limited upgrade potential compared to the rate of evolution of stand-alone MR systems. The ability to insert the brain PET into the latest MRI platform gives it substantial flexibility in integrated performance over the hybrid integrated whole-body approach where the MRI technology cannot keep pace.

Based on a need for 5,902 brain PET scanners by 2020, if we assume a ramp up time of 10 years and a product life of 10 years, a reasonable estimate of sales per year would be 600 units. If each unit sold for $1 million USD, gross sales per year would be $600 million. Adding in a service contract cost of 10% of sale price would generate annual revenue of $100,000 per unit sold. After 10 years, service contract revenue would total $600 million annually, resulting in total annual revenues of $1.2 billion USD.

## LIMITATIONS

Screening of those at risk was set to once per year but those with a negative screen might not have to be re-scanned for several years, significantly reducing the number to be screened. (Note that this would increase prevalence above 0.1 increasing expected value.) If, for example, screening was reduced on average to once every two years, then the total number of brain PET units required could be reduced to 300 per year. This would still justify development of a brain PET imaging system especially given the fact that other diseases/conditions would also benefit from the use of the brain PET scanner, thus increasing demand for the product.

The calculation of the expected value is dependent on the assumed value of QALY and willingness to pay (WTP) which was taken from the Swedish/Finnish population [44]. If WTP was significantly lower than $80,000 USD (2011 dollars) then the expected value might become negative. Also, the expected value would become smaller and possibly negative if delay of onset was less than five years.

The expected value, as calculated by equation 1, is inversely dependent on the cost of treatment which we have estimated at $24,000 CAD per person total over five years. This may be an overestimate. If we use the incidence figures for the USA as given in Table 5, treating the 255,000 individuals identified each year for 10 years at a cost of $48,000 CAD would cost $12.24 billion for the first cohort alone. This may be high as compared to other treatments for conditions with high incidence (e.g., treatment of high cholesterol with statins) where the cost is much lower. Hence the expected value using this estimated treatment cost may be underestimated.

Note that our estimates for expected value do not apply to any specific jurisdiction since values used come from the literature and are associated with different countries (QALY, WTP, treatment costs, value of delayed onset, technology costs). However, our analysis provides a starting point for a complete traditional cost effectiveness analysis for a specific jurisdiction.

In our estimate of the costs of screening by brain imaging we have made a number of assumptions. First, that combined PET/MRI represents an upper limit to equipment costs since it is possible that MRI alone or PET alone could be sufficient for screening as new imaging biomarkers are discovered. Second, that the assumption of needing only one PET probe which can be labeled with ^18^F (allowing for both production and remote delivery) represents a lower limit to radiopharmaceutical costs. Third, that this PET probe will have a Se and Sp of 0.9 when combined with MRI. In future work it would be instructive to investigate the variation in the expected value for existing PET probes such as glucose-PET, amyloid-PET, and tau-PET and for new ones as they are discovered.

## SUMMARY

We propose that there is healthcare economic justification to screening by brain PET/MRI for individuals who may develop dementia due to lifestyle choices. We also argued that a high-resolution MRI-compatible brain PET insert is a commercially viable diagnostic imaging device that will assist in detecting dementia and delaying its onset.

We propose that screening by advanced imaging for dementia is needed: 1) to target what could be expensive therapy only to those that have a high probability of developing cognitive impairment, 2) to allow the evaluation of therapy through repeat imaging when explicit symptoms are largely absent, and 3) to provide a powerful incentive to change lifestyle.

## CONFLICT OF INTEREST

The authors have no conflict of interest to report.
